# Supplementary material for: The impact of a school garden program on children’s food literacy, climate change literacy, school motivation, and physical activity: A study protocol
Source: PLoS One. 2025 Apr 24;20(4):e0320574. doi: 10.1371/journal.pone.0320574 (PMC12021283; doi:10.1371/journal.pone.0320574)
Supplement: S1 File — (DOCX) [file pone.0320574.s001.docx]

**
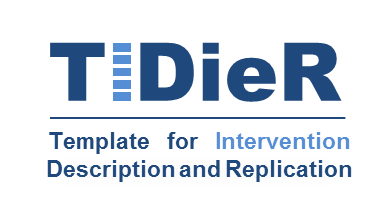
**

The FoodACT Intervention TIDieR checklist

1. **Brief name**FoodACT
2. **Why**The FoodACT project evaluates a school garden program delivered by the Danish organization Haver til Maver (Gardens to Bellies (GtB))*.* The GtB school garden program was established in 2006 and is currently delivered in nine municipalities across Denmark.

The program primarily targets pupils in 4^th^ grade, who attend eight sessions (on some occasions in selected municipalities, ten sessions) in the school gardens during the last semester of 4^th^ grade and the first semester of 5^th^ grade. However, GtB also delivers tailored programs to kindergarten children, children with special needs, and adolescents. The program builds upon a facilitative approach in which the pupils are actively engaged in educational activities in the school gardens a location away from school plots. The activities include weeding, seeding, harvesting, and preparing meals from the pupils’ own produce. The activities in the school gardens aim to promote critical and practical skills, tasting and quality of organic and seasonal produce, pupil-pupil interactions and interaction with the environment, and an active use of all senses (Wistoft, 2013). Activities are characterized by genuine participation by combining hands-on, experiential methods for active participation with a focus on sensory and social processes (Jensen & Simovska, 2005). The didactic setting of the school gardens thus provides unique hands-on opportunities for pupils to develop their knowledge, skills, and motivations in relation to health, well-being (Malberg Dyg & Wistoft, 2018), learning, and sustainability. Through this, it is expected that the program contributes to developing the pupils’ food literacy, climate change literacy, motivation for participating in school activities, and physical activity.

The FoodACT logic model illustrates the potential mechanisms and effects of GtB on participating pupils in relation food, sustainability, school motivation and physical activity. In this way, school gardens are prosed to provide a motivating school setting that promotes learning and critical competences through an authentic nature-based setting. For more information, see the FoodACT protocol.


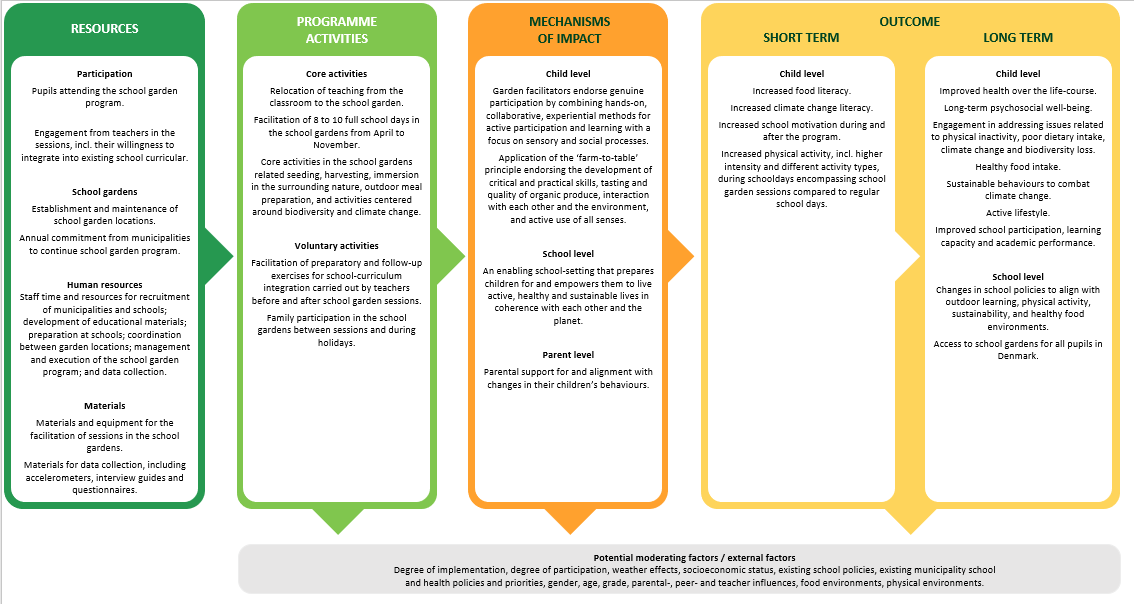


The FoodACT Logic model

1. **What (materials)**All garden facilitators are trained in relevant professions such as the culinary arts, biology, or gardening. Upon employment in GtB, they are further trained in GtB’s tailored eight (on some occasions in selected municipalities, ten) session program, which is similar in content across the garden locations, but which is delivered with slight alterations according to the unique physical setting of each school garden. The garden facilitators are further trained in the specific didactic and pedagogical approach employed by GtB. During the school garden season, the facilitators are provided with the necessary gardening and cooking equipment for the execution of each session.

Prior to the beginning of the program, schoolteachers are provided with a poster illustrating the thematic content of each school garden session. Additionally, the teachers have access to an online GtB platform with video-material and other teaching materials (all in Danish) that aim to inspire integration of educational content from the school gardens into the existing school curriculum.

1. **What (procedures)**Prior to the beginning of the program, information meetings are held with each participating school class, at which the garden facilitator introduces the outline of the program and its thematic contents. The meeting prepares teachers and assisting pedagogues to the program, the academic goals of the educational activities, and how GtB expects the pupils to benefit from the program. Moreover, an introductory workshop (1-2 hours) is conducted with the pupils to spike their curiosity before the first school garden session. In this workshop, the pupils are introduced to, amongst others, different seeds, plants and the process of photosynthesis. They also receive a large poster to hang in the classroom. The poster is colorful and shows the program the pupils will get engaged in during their participating in GtB.

In the school gardens, activities relate not only to the practical aspects of growing and preparing foods, but also incorporate more theoretical elements related to climate change, biodiversity, and organic approaches to food production. The overarching purpose is for pupils to learn where their food and produce comes from, how to take care of crops, and how to prepare meals with the vegetables and fruits they grow in the garden or find in the surrounding nature.

1. **Who provides**All activities in the school gardens are facilitated by experienced GtB garden facilitators. A responsible teacher (1-2 per class) from each participating school class attends the school garden sessions together with the pupils, and are encouraged to support the garden facilitators during the sessions, as well as to integrate the themes and learnings in the gardens into their regular school curriculum.

   The GtB program has existed for more than 18 years, and the organization therefore has thorough experience with its delivery, as well as a good existing collaboration with both recurring and new schools.
2. **How**Activities in the gardens are led by the GtB-trained garden facilitators. Pupils are divided into smaller groups, who are assigned a plot of land. Throughout the various sessions, the pupils become responsible for preparing, weeding, seeding, and harvesting the produce in their plot. Each session has a specific theme with related activities such as seasonal fruits and vegetables, wild herbs, raw foods, decomposition, biodiversity, and sustainable meals. Activities encourage the pupils to explore, sense, experiment, take leadership, collaborate and play with each other and the knowledge they gain in the school gardens. Moreover, physical activity plays a central role in the school garden through bodily work such as digging, lifting, and watering the plot.
3. Where
   The GtB program is currently delivered in nine different school gardens located in the municipalities of Fredensborg, Gentofte, Hørsholm, Vordingborg, Frederiksberg, Roskilde, Gribskov, Odense, and Svendborg.

   All participating school classes attend a school garden located in their own municipality, but the distance to the garden may vary across schools within the same municipality. School classes often transport themselves to the garden either by bike, walk or public transport (or a combination).
4. **When and how much**The school garden program varies slightly between the garden locations, but typically consists of four sessions of four hours during the school day (from 9.00-13.00) in the spring (April-June) and four sessions of four hours during the school day in the autumn (August-October).

   In one of the current municipalities that has incorporated GtB and where it is mandatory for public schools to be enrolled in the GtB program, there is not enough space in the garden for all classes to have full school days in the school garden. Therefore, these classes are attended five sessions of two hours (either morning from 9.00-11.00 or afternoon from 11.30-13.30) in the spring, and three sessions of two hours (either morning from 9.00-11.00 or afternoon from 11.30-13.30) during the autumn.

During school holidays, pupils and their families are encouraged to water the vegetables in the school gardens by themselves. Moreover, teachers are encouraged to integrate educational activities and themes from the school garden into the regular school curriculum. Due to the voluntary nature of these components, the exposure of these will vary across schools and pupils.

1. **Tailoring**

N/A

1. **Modifications**N/A
2. **How well (planned)**

The FoodACT study aims to evaluate the GtB program through a multimethod, quasi-experimental inquiry. The overarching aim will be to investigate the impact and mechanisms of the GtB program on pupils’ food literacy, climate change literacy, and general school motivation. Food literacy, climate change literacy and general school motivation will be measured in the intervention and control group using pre- and post-intervention surveys. Physical activity will be assessed using accelerometery, and acute school motivation by text-message surveys only in the intervention group. Semi-structured interviews and focus-groups will be held with garden facilitators and pupils in the intervention group to explore the implementation of the program and the mechanisms behind the promotion of food literacy and climate change literacy. Results are expected to be published by 2027.

1. **How well (actual)**

N/A

**References**

Jensen, B. B., & Simovska, V. (2005). Involving students in learning and health promotion processes—Clarifying why? What? And how? *Promotion & education.*, *12*(3–4), 150–156. https://doi.org/10.1177/10253823050120030114

Malberg Dyg, P., & Wistoft, K. (2018). Wellbeing in school gardens – the case of the Gardens for Bellies food and environmental education program. *Environmental Education Research*, *24*(8), 1177–1191. https://doi.org/10.1080/13504622.2018.1434869

Wistoft, K. (2013). The desire to learn as a kind of love: Gardening, cooking, and passion in outdoor education. *Journal of Adventure Education and Outdoor Learning*, *13*(2), 125–141. https://doi.org/10.1080/14729679.2012.738011
