# Supplementary material for: The impact of a school garden program on children’s food literacy, climate change literacy, school motivation, and physical activity: A study protocol
Source: PLoS One. 2025 Apr 24;20(4):e0320574. doi: 10.1371/journal.pone.0320574 (PMC12021283; doi:10.1371/journal.pone.0320574)
Supplement: S3 File — (DOCX) [file pone.0320574.s003.docx]

Study title:

**FoodACT:**

Investigating the impact of a school garden intervention on children’s food literacy, climate literacy, physical activity and school motivation.

**Study synopsis**

Version 1.0

January 2023

# Study centre / delivery details

Center for Clinical Research and Prevention.

**Investigators**

| Peter Elsborg | Researcher, PhD | Center and Clinical Research and Prevention (CCRP), Bispebjerg and Frederiksberg Hospital |
| --- | --- | --- |
| Mads Bølling | Researcher, PhD | Center and Clinical Research and Prevention (CCRP), Bispebjerg and Frederiksberg Hospital |
| Peter Bentsen | Director and affiliated professor | Center for Clinical Research and Prevention (CCRP), Bispebjerg and Frederiksberg Hospital and Department of Geosciences and Natural Resource Management (IGN), University of Copenhagen |
| Glen Nielsen | Associate Professor | Department of Nutrition, Exercise and Sports (NEXS), University of Copenhagen |
| Anna Stage Hansen | Research assistant, PhD-student on the project | Clinical Research and Prevention (CCRP), Bispebjerg and Frederiksberg Hospital, and Department of Nutrition, Exercise and Sports (NEXS), University of Copenhagen |
| Marie Caroline Vermund | Research assistant | Clinical Research and Prevention (CCRP), Bispebjerg and Frederiksberg Hospital |

**Principal investigator**

| Peter Elsborg | Researcher, PhD | Center and Clinical Research and Prevention (CCRP), Bispebjerg and Frederiksberg Hospital |
| --- | --- | --- |

**Project coordinator**

| Anna Stage Hansen | Research assistant, PhD-student on the project | Clinical Research and Prevention (CCRP), Bispebjerg and Frederiksberg Hospital and Department of Nutrition, Exercise and Sports (NEXS), University of Copenhagen |
| --- | --- | --- |

**Other staff**

| Camilla Roed Otte | PhD, Development manager | The organization Haver til Maver |
| --- | --- | --- |
| n.n. | Student Assistant | Clinical Research and Prevention (CCRP), Bispebjerg and Frederiksberg Hospital |
| n.n. | Student Assistant | Clinical Research and Prevention (CCRP), Bispebjerg and Frederiksberg Hospital |

**Synopsis FoodACT study**

# Study Objective

The objective of this study is to investigate how the school garden intervention ‘Haver til Maver’ influence 4^th^-5^th^ grade pupils food literacy, climate literacy, physical activity, and school motivation.

The objective is research through three sub-studies:

- - *Sub-Study 1 (SS1):* Investigation of the efficacy of the ‘Haver til Maver’ intervention on 4^th^- 5^th^ grade pupils’ food literacy, climate literacy, and school motivation.
  - *Sub-Study 2 (SS2):* Investigation of the ‘Haver til Maver’ interventions influence on 4^th^-5^th^ grade pupils physical activity.
  - *Sub-Study 3 (SS3):* Investigation of the contextual characteristics of gardening and the 4^th^-5^th^ grade pupils experience of the ‘Haver til Maver’ intervention.

# Overall Study Design

Schools attending the ´Haver til Maver’ intervention will be selected as the intervention group and will participate in all three sub-studies. Schools not attending the ‘Haver til Maver’ intervention will be recruited and invited to answer questionaries about their food literacy, climate literacy and school motivation and will act as a control group for Sub-Study 1.

The efficacy study (SS1) will quantify the effects of the ‘Haver til Maver’ intervention using a pre- post quasi-experimental design measuring the pupils’ food literacy, climate literacy and their school motivation before and after the intervention.

The second study (SS2) will quantify the influence of the ‘Haver til Maver’ intervention using a within subject design on the pupils’ physical activity at two weeks long sessional periods during the intervention.

The third study (SS3), a process evaluation, will investigate the contextual characteristics in the garden using systematic observations and investigate the pupil´s experience of the ‘Haver til Maver’ intervention with focus-groups interviews using thematic content analyses.

Pilot-study: Initially, a pilot-study will be conducted with the purpose to test and modify the selected SS1-3 research methods: to inspect and adjust implementation of the questionaries about food literacy, climate literacy, and school motivation. In addition, the purpose is to validate the SENS motion® accelerometer by investigating whether this instrument is valid to measure children’s physical activity. The investigation of the validity of the SENS Motion® accelerometer is conducted by comparing the measurements to the commonly used Axivity® AX3 accelerometer. If

the SENS Motion® accelerometer is valid for the target group, this accelerometer will be used in Sub-Study 2. The third purpose is to evaluate the PARAGON observation method.


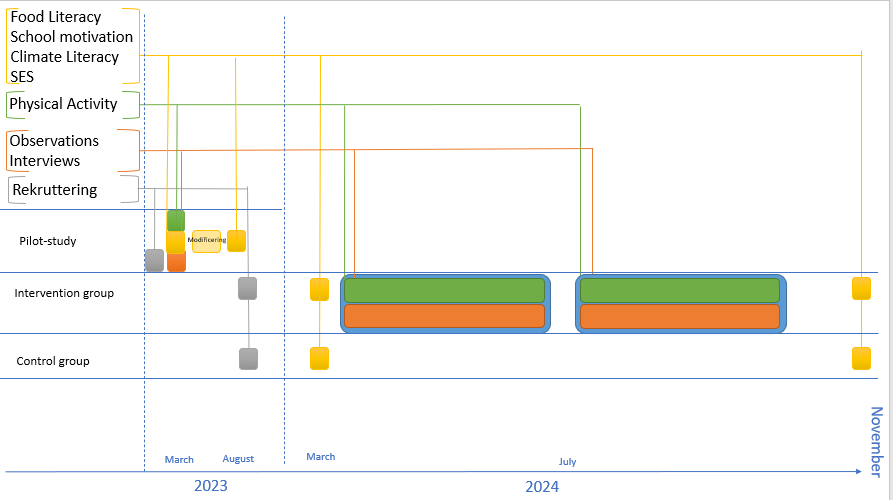


Figure 1: Food-ACT study design and timeline.

# Participants

The participants are pupils from 4^th^-5^th^ grade (age 10-12) from Danish municipal primary and lower secondary schools from municipals of Region Zealand, Region Southern Denmark and The Capital Region. The study aims to recruit schools within municipalities that already have ‘Haver til Maver’ school gardens in their district (intervention group) and schools in municipalities that does not (control group). In both groups special attention will be given to recruit schools with pupils from low socio-economic areas.

The study has two different participant samples. In Sub-Study 1 the participants are pupils from 4^th^- 5^th^ grade from Danish municipal primary and lower secondary schools attending the ‘Haver til Maver’ intervention (intervention group) and pupils from 4^th^-5^th^ grade from Danish municipal primary and lower secondary schools not attending the ‘Haver til Maver’ intervention (control group). In precent year, 2023, pupils from 142 school classes in Denmark participated in the intervention. We expect to recruit proximately 990 pupils for SS1 for both intervention and control group.

In Sub-Study 2 and Sub-Study 3, the participant sample is a subsample of the intervention sample in SS1. The participants are pupil’s 4^th^-5^th^ grade (n=540) from 30 Danish municipal primary and lower secondary schools within the listed regions.

The 30 schools are purposely sampled among schools in municipals of Region Zealand and Region Southern Denmark, the Capital Region with a higher proportion of citizens having a lower level of education compared with region averages.

The estimated drop-out during the intervention is 7% of classes, corresponding to two classes. In total, approximately 990 pupils with consent to participate will be enrolled. In each of the enrolled classes, the estimated drop-out during the intervention is 30% corresponding to 7 pupils.

## Inclusion Criteria

- 4^th^-5^th^ grade school classes in Danish municipal primary and lower secondary schools participating in the ‘Haver til Maver’ intervention.
- Classes not involved in other school development or research projects.
- Participants with parents (or legal guardian) having provided written informed consent.

## Exclusion Criteria

- Pupils with significant health problems as judged by the investigators will be excluded from the analysis (Sub-study 1-3).

# Study procedures

Upon teacher content to participate, the investigators will visit each of the participating classes two times during 2023 (pilot-study) and two times in 2024, the year where the intervention takes place. Before each visit, teacher-assisted communication to the parents (or legal guardian) or legal guardians is performed, informing them about their child’s participation in the study. By assistance from the teachers, the informed consent (a physical document) is provided to the parents (or legal guardian) or legal guardians and is returned to the researchers at the day of the first visit.

The children complete a food literacy, climate literacy and school motivation questionnaire in the classroom during school hours administrated by the classroom teacher before and after the ‘Haver til Maver’ intervention. The full procedure is estimated to last 2x 45 min. The accelerometer is worn by the pupil in fourteen days, before being collected by an investigator visiting the classes.

At each school visit, two investigators (one male and one female) invite four pupils at the time to a room next to the classroom. The male researcher handles male participants’, and the female researcher handles female participants. In the room, by turn, 1) SENS motion® accelerometers are mounted with plaster on the right thigh or 2) Axivity® AX3 accelerometers (23 x 32.5 x 7.6 mm, are mounted with skin tape (with the following brands: Fixomull tape (BSN Medical), adhesive hair-set tape (3M, USA), Opsite Flexifix (Smith & Nephew, UK)) by the investigator to each of the pupils’ left thigh. The pilot-study will decide whether the SENS motion® or Axivity® AX3 accelerometers will be used to measure physical activity in Sub-Study 2.

During the intervention period, pupils in the intervention group will have their session-activity measured during two ‘Haver til Maver’ sessions, while the pupil’s wear the accelerometers, by using The Physical Activity Research and Assessment tool for Garden Observation (PARAGON).

# Measurements

No biological samples will be obtained from the participants in this study.

From the pupils, only data on devise-based measured physical activity (accelerometers), as well as self-reported school motivation food literacy and climate literacy is collected. Additionally, onsite researcher observations data of physical movements, postures and motion, and the interview data on pupil’s experience with the intervention is collected.

PARAGON is a systematic and valid observation tool capturing the contextual characteristics of gardening. Upon observations, a random selection of children will be invited to participate in a focus group interview converging their experience with the ‘Haver til Maver’ gardening activities.

From the pupils’ parents (or legal guardian) data on family socio-economic-status (parent or legal guardian education level and civil status) is collected.

# Outcomes

*Sub-study 1:* The outcomes are the pupil’s food literacy, climate literacy, and school motivation. To investigate the effect of the ‘Haver til Maver’ intervention on food literacy, climate literacy, and school motivation, the pupils will answer the questionaries before and after the intervention. Linear mixed models will be applied to assess the intervention effects on food literacy, climate literacy, and school motivation. The analyses will account for clustering of pupils in school classes and schools as well as known confounders such as gender, age, and SES.

*Sub-study 2*: The primary outcomes are pupils’ physical activity i.e., sedentary behaviour (SED), light physical activity (LPA), moderate-to-vigorous physical activity (MVPA).

*Sub-study 3*: The primary outcomes are the pupil’s movements, postures, motions, and experience of the intervention activities. When using systematic observations with PARAGON pupils’ movements and motions will be captured when they attend the gardening intervention activities. The observed movements and motions are categorised into four categories: overall PA-level, garden-related tasks, garden related motions, social associations, and interaction.

Semi-structured focus-group interviews will be held after a day with gardening intervention activities with both the involved pupils, teachers, parents (or legal guardian) and gardens facilitators. The thematic content analyses will be open to unforeseen themes that may emerge while also keeping a focus on the different target groups’ motivation and experience with the intervention.

# Reimbursement

Each of the participating classes will receive a taxable reimbursement of 1.500 DKK to use in the bank account of the class (klassekassen).

# Timeline

The data collection of the pilot-study is planned to start in March 2023 and end in November 2023. The data collection of the Sub-Study 1-3 is planned to start at March 2024 and will be finalized within November 2024, if no unforeseen incidences happen. The post-data collection operations and analyses, publication etc. is expected to end by December 2025 (please see figure 1).

# Datahandling

All data and other personal information that is collected during the study period is processed in accordance with the Danish Data Protection Act, i.e., it will be confidentially stored in secured systems only accessible by persons who are affiliated with the study and who therefore have a duty of confidentiality. Names of pupils and parents (or legal guardian) will not appear on any material

(except consent forms), which will instead be affixed to a participant number. Likewise, names of teacher, pupils, parents (or legal guardian), and garden facilitators etc. will not appear in either reports or other publication of results from this study.

# Risks of study participation

A small number of pupils, corresponding to approx. 5 out of 100, may experience skin irritation when having the Axivity® AX3 accelerometers mounted to the skin with skin-tape or the SENS Motion® accelerometer mounted with plaster. The participating pupils’ and their parents (or legal guardian) are informed to de-mount, at store the accelerometer, if this should occur.

There may be risks that we are not currently aware of. Participants will therefore be asked to inform the PI if they experience health problems during the study. If we discover side effects that we were not already aware of, the subjects will be informed immediately, and the PI responsible for the study will decide whether they can continue in the study.
