# Supplementary material for: The impact of a school garden program on children’s food literacy, climate change literacy, school motivation, and physical activity: A study protocol
Source: PLoS One. 2025 Apr 24;20(4):e0320574. doi: 10.1371/journal.pone.0320574 (PMC12021283; doi:10.1371/journal.pone.0320574)
Supplement: Attachment — Submitted filename: Response to Reviewers.docx [file pone.0320574.s005.docx]

| Reviewer #1: | Thank you for taking the time to review our manuscript. We greatly appreciate your thoughtful comments, all of which have been addressed and elaborated upon in detail below. |
| --- | --- |
| The citation in the text is to be separated from the word e.g. performance(1–3) to be presented as performance (1-3). This applies throughout the manuscript. | Thank you for spotting this. We have now corrected this throughout the manuscript. |
| Line 220-221: SS2 and SS3 to be spelled out as sub-study 2 and sub-study 3 or SS is to be denoted/indicated in Table 1. | We agree and have now denoted the three sub-studies in Table 1. |
| Line 227-231: the detailed sampling process/procedure is to be described. | Thank you for giving us the chance to clarify. In the new version of the manuscript, we have elaborated on how the participants were selected for the intervention and control group.  Line 228-231 p.10: “*Municipalities decide whether to offer the GtB program to schools within their district, which are then signed up for the program on a yearly basis. All contact information for the schools will be provided by GtB.”*  Line 239-247 p. 11: *”First, a list will be comprised of municipalities that approximately match intervention municipalities on average disposable income (based on 2017 data from Statistics Denmark)(Engmann, 2019) and are in the closest possible geographic proximity to the intervention municipality. For example, intervention schools located on the Danish Island of Funen will be matched with control schools in a different municipality on Funen but with a similar municipal average disposable income. Based on this list, public (i.e. non-private) schools within suitable control municipalities will be invited to participate, and an even distribution of school classes within the matched municipalities will be attempted. However, all schools indicating that they wish to participate will be enrolled in the study. “*  Line 259-261 p. 11: “*The study will strive to include all schools participating in GtB in 2024 in the study’s intervention group with no upper limit for participation. Sampling for the control group will aim to match the number school classes in the intervention group.”* |
| Line 244: the language version of the questionnaires/inventories/surveys/tools used in the study is to be clearly stated. | We agree and have now added that all questionnaires were administered in Danish. |
| Line 276: the classification for SOSC is to be provided e.g. Class I to Class VI. | In the new version of the manuscript, we have added that the SES were classified from class I to Class VI and further explained the procedure for these classifications. We refer to the Danish Occupational Social Class coding-scheme for further information. Please see line 351-360 p. 15. |
| Line 284: define what is home economics. | Thank you for giving us the chance to clarify. Based on the various comments regarding home economics, we have realized that translating ‘madkundskab’ (which translates directly into something like ‘food science and competence’) into home economics is confusing and hence can be misleading. We have changed it to ‘food and cooking classes’ throughout the manuscript, which hopefully clarifies its meaning. |
| Line 274-296: the classification of categories (categorical) data of these variables is to be provided e.g. Variable A 1= , 2= 3= , 4= | We have elaborated the use of all our covariates including the classifications of categorical data. |
| Similarly, for the outcome measures, how FL, CCL, and SM will be classified and analysed e.g. scale or categorical data. For categorical data, if possible the coding for the stems is to be included. This applies for other tools in Line 336-359. | In the new version of the manuscript, we have elaborated how they were classified and analysed for FL, CCL and SM in the 2.4.2 section starting at line 298-343 p. 13-14.  Additionally, this has been elaborated for section 2.5.2 starting at line 481-530 p. 21-22.  Moreover, we have moved the covariates which is only used in the PA analysis to this section, so it does not confuse the reader. |
| Line 303-306: the sentence is not clear and requires revision. Need to state clearly how many classes and how many students in a class required for the sample size.  Line 305: the power is to be stated. | Thank you for the observation and comment. We agree, and have revised the line, 389-393 p. 17 which hopefully clarifies the procedure for calculating the sample size for a minimal detectable effect size. The sample size was calculated prior to recruitment and was based on how successful previous related studies conducted in a Danish setting had been in their recruitment. Since no similar studies estimating the effect of a similar intervention on e.g., our FL measure had been conducted, we calculated the sample size based on expected recruitment rather than expected effect size. We therefore used the expected sample estimate of 25 school classes with 18 pupils on average in a Danish school class to calculate a minimal detectable effect size (Cohen’s d). This resulted in a minimum of 450 pupils in each group, and we used this estimate as a minimum target for our recruitment.  The power calculations provided us with a minimum recruitment goal of 900 pupils and did thus not provide an upper limit for our recruitment. In practice, we aimed at recruiting all school classes participating in the GtB program in 2024. Based on this, we aimed at recruiting a similar number of school classes for the control group. We did therefore not limit ourselves to the minimum recruitment goal set by the power calculations, and instead used the logistical opportunity (rather than constraint) provided to us by the fact that the GtB intervention already had a high number of school classes registered for the program.  Line 402-403 p.17 states the power used for the calculations. |
| For SS1 and SS2: Were the outcome variables simultaneously or independently analysed?  Multiple testing correction is to be applied where required. | We had specific hypothesis related to each outcome; therefore, the outcome variables were analysed independently and multiple testing correction were applied where required. |
| Line 319: SES and home economics may be collinear. If collinearity is present, it should be addressed accordingly. | Thank you for this comment, and the chance to clarify. Your comments have made us aware that translating ‘madkundskab’ (which translates directly into something like ‘food science and competence’) into home economics is confusing and hence can be misleading. We have changed it to ‘food and cooking classes’ throughout the manuscript, which hopefully clarifies its meaning.  Since the variable ‘food and cooking classes’ refers to food and cooking education at a school class-level (yes/no), and is based on national school curricula regulation, we do not expect any collinearity with SES as this is measured at the individual level. |
| Line 322: the statistical software, its version and publisher name is to be stated. | We have further clarified our use of statistical software in line 426-428 p. 18: “*All analyses will be conducted using RStudio (version 1.4.1106; RStudio, Inc.) and IBM SPSS Statistics (version 29.0.1.0; IBM Corp.).*” |
| Line 343: typo ‘school garden sessions: Will’ | This is now corrected. |
| Line 361: how the sampling is done to be clearly stated. | We have further elaborated on the sampling procedure for SS2 in line 533-537 p. 22.  Additionally, we corrected the intended number of participating classes.  *“In SS2, a subsample of the intervention group containing 45-55 classes will be recruited, based on the former estimate calculation described in section 2.4.4 This number of classes was chosen in terms of resources, practicality, and distance to the school gardens. However, school gardens from both rural and urban areas were included securing representativity in the sub-sample.”.* |
| Line 364-365: the sentence is unclear. More information is to be provided. | In the new version of the manuscript, we have rephased the sentence. Please see line 539-541 p. 22: “*Including the minimum of 45 classes with an estimated average of 18 pupils per class will provide 11.340 days with PA data from accelerometer based on 810 children since data is based on seven days with two measurements”.* |
| Line 374: the reason for these variables and not others were controlled is to be stated. | We have elaborated this in line 345-351 p. 15  *“The inclusion of confounders described in this section are based on previous literature [1,18,26,27]., Socio-economic status (SES), gender and age will be used in all analyses. Food and cooking class participation will be included in analyses related to FL., Owhereas the other covariates, such as the related school activities, transport, weather, and the size of the school garden will be included used in analyses relevant to FL (only home economics) and PA”.* |
| Line 416: typo tour | Thank you. This is now corrected. |
| Line 438: the version of the software is to be stated. NVIVO is to be written as NVivo. | Thank you. This is now corrected. |
| Not all references conformed to the journal format. | Thank you. We have made sure that all references conform to the journal format. |
| **Reviewer #2:** | Thank you for your time and effort in reviewing our manuscript. We greatly appreciate your valuable comments. In particular, the opportunity to discuss the rationale for a quasi-experimental design over a RCT. |
| * I would encourage you to provide a more nuanced discussion of the rationale for a quasi-experiment over an RCT in your context. It is fair to say that RCTs (often) prioritise internal over external validity, but establishing internal validity is important as a pre-condition for understanding external validity. I would encourage you to discuss why external validity is the priority for this particular intervention and study at this particular time, given the existing evidence base. | Thank you. In the new version of the manuscript, we have described more clearly, why we prioritized pragmatic validity (in terms of implementation and scalability) over internal validity by choosing to study a school-garden program already in place and in use in municipalities, which hindered an RCT approach.  The following has been added in line 156-164 p. 7:  “*The aim of the FoodAct study was to study the impacts of an already established school-garden program, namely the GtB program. This was chosen in order to ensure scalable and sustainable implementation of the intervention studied which is important for pragmatic validity and practical/political relevance of the study. The GtB program is implemented at the municipality level, which means that it is a political decision to financially prioritize whether the schools within the municipality get to participate in GtB or not. Randomizing which schools or classes would get to participate is thus not acceptable from a municipal perspective (in Denmark) making it impossible to* *recruit municipalities and hence schools to an RCT trial of this school garden program.* “  Prior to this description, we have also added that a previous RCT study has shown effect of school garden. In the strengths and limitations section, we have already described the limitations it entails not to applying an RCT design. |
| * Accepting, however, that a clustered quasi-experimental design is the feasible design of this study, the credibility of the results will be much stronger if it is clearly explained how the comparison schools were recruited to maximise the comparability across intervention and comparison groups. The current statement that “recruitment and inclusion of control classes will be based on similarity with the intervention schools regarding geographical proximity and average disposable income at the municipal level” is rather vague. What does it actually look like in practice? | In the new version of the manuscript, we have further elaborated on the sampling procedure for control classes in line 228-231 p. 10: *“Municipalities decide whether to offer the GtB program to schools within their district, which are then signed up for the program on a yearly basis. All contact information for the schools will be provided by GtB.”*  Line 239-247 p. 10-11: *”First, a list will be comprised of municipalities that approximately match intervention municipalities on average disposable income (based on 2017 data from Statistics Denmark) (Fakta Om Indkomster Og Formue - Danmarks Statistik, 2019) and are in the closest possible geographic proximity to the intervention municipality. For example, intervention schools located on the Danish Island of Funen will be matched with control schools in a different municipality on Funen but with a similar municipal average disposable income. Based on this list, public (i.e. non-private) schools within suitable control municipalities will be invited to participate, and an even distribution of school classes within the matched municipalities will be attempted. However, all schools indicating that they wish to participate will be enrolled in the study.“*  Line 259-261 p. 11: *“The study will strive to include all schools participating in GtB in 2024 in the study’s intervention group with no upper limit for participation. Sampling for the control group will aim to match the number school classes in the intervention group.”* |
| * You set out sample sizes in lines 227-228 but it is not clear (at least at this point) why these particular sizes are chosen. They don’t seem to be based on power calculations (since these are reported later and use different, more conservative, values), so is it logistical constraints, or something? | The sample size was calculated prior to recruitment and was based on how successful previous related studies conducted in a Danish setting had been in their recruitment. Since no similar studies estimating the effect of a similar intervention on e.g., our FL measure had been conducted, we calculated the sample size based on expected recruitment rather than expected effect size. We therefore used the expected sample estimate of 25 school classes with 18 pupils on average in a Danish school class to calculate a minimal detectable effect size (Cohen’s d). This resulted in a minimum of 450 pupils in each group, and we used this estimate as a minimum target for our recruitment.  The power calculations provided us with a minimum recruitment goal of 900 pupils and did thus not provide an upper limit for our recruitment. In practice, we aimed at recruiting all school classes participating in the GtB program in 2024. Based on this, we aimed at recruiting a similar number of school classes for the control group. We did therefore not limit ourselves to the minimum recruitment goal set by the power calculations, and instead used the logistical opportunity (rather than constraint) provided to us by the fact that the GtB intervention already had a high number of school classes registered for the program. |
| * Background descriptive measures are extremely vaguely specified and, given these are proposed to be included in the model for estimating impact, should be much more explicit to ensure there is less ambiguity in that modelling. At the moment there would be huge variation in the models you could actually run to estimate the impact of the study, which may lead to differing conclusions and, hence, undermines the benefits of having pre-registered this study with this protocol. | We have elaborated all our background descriptives with information on how they were collected, and the data type (scale, categorical, or binary). Additionally, the have added a sentence of how we intend to use the confounders in our analysis. Please, see line 345-351 p. 15.  *“The inclusion of confounders described in this section are based on previous literature (1,18,27,28). Socio-economic status (SES), gender and age will be used in all analyses. Food and cooking class participation will be included in analyses related to FL., other covariates, such as the related school activities, transport, weather, and the size of the school garden will be included in analyses relevant to PA.* |
| * On the models themselves (sections 2.4.5 and 2.5.4), it would make the model you propose to run much clearer to write it down algebraically. This interplays with the above suggestion to be far more explicit about the other covariates that are being included in the model. | Thank you for your suggestion. We have included the model equations in the proposed sections. Please see section 2.4.5 and section 2.5.4.   Additionally, based on your comment, we have decided to move the covariates only used in SS2 down to the section describing the second study. |
| * You sensibly say that you will estimate ICCs but do not say how. Will you extract these directly from your main impact estimation model? Some methodological literature instead advocates use of a model without the various covariates to estimate the ICC, so your current plan is ambiguous. | The procedure has been clarified in the text on line 423-424: “*Lastly, ICCs will be calculated by running the linear mixed model without the covariates to assess potential variation among school classes as fractions of the total variance*.” |
| Minor points  * Line 218: invented should be invited? | Thank you, this has been corrected. |
| * Line 314: factor should be intercept? | This is corrected in the new version of the manuscript. We have used the term ‘random effect’ instead of intercept. |
| * Line 321-322: “two-tailed tests with p-values below 0.05 will be considered statistically significant.” Is this meant to come earlier (~ line 316) to be about testing the coefficient on the focal interaction term. | We have moved this up to line 411, as suggested. |
| * Line 361: in “containing of” the “of” is superfluous | Thank you. We have deleted ‘of’ now. |
| * There are various other typos and the manuscript would benefit from a check to eliminate these, especially because some do result in ambiguity of meaning. | Thank you. We have corrected for typos throughout the manuscript. |
